# Coexistence of increased arterial stiffness and interatrial block in overweight subjects

**DOI:** 10.1111/anec.12724

**Published:** 2019-11-10

**Authors:** Mustafa Dogdus, Goksel Cinier

**Affiliations:** ^1^ Department of Cardiology Training and Research Hospital Usak University Usak Turkey; ^2^ Department of Cardiology Kackar State Hospital Rize Turkey

**Keywords:** clinical, epidemiology/clinical trials, noninvasive techniques—electrocardiography

## Abstract

**Background:**

Interatrial block (IAB) is an electrical conduction delay between the right and left atrium and is associated with some cardiovascular disorders. Arterial stiffness is a useful prognostic marker for cardiovascular events. In the present study, we aimed to investigate the coexistence of increased arterial stiffness and IAB in overweight subjects.

**Methods:**

A total of 110 overweight people were enrolled (56 subjects with IAB, and 54 age‐ and gender‐matched subjects without IAB) into the study. Surface 12‐lead standard ECGs were recorded. I.E.M. Mobil‐O‐Graph ambulatory blood pressure monitor device was used to assess the arterial stiffness.

**Results:**

The mean age of the patients was 54.1 ± 11.5 years, and 53.6% were male. PWV and Aix were significantly higher in IAB (+) group than IAB (−) group (9.34 ± 1.5 vs. 7.86 ± 1.3, *p* < .001; 29.18 ± 11.2 vs. 22.75 ± 10.4, *p* < .001, respectively), and also, positive linear correlation was observed between arterial stiffness parameters and P‐wave duration (*r* = .758 for PWV; *r* = .682 for Aix, respectively).

**Conclusion:**

The present study is the first to focus on evaluating the relationship between the presence of IAB and arterial stiffness in overweight subjects. If there is a coexistence of increased arterial stiffness and IAB in overweight subjects, it should be considered as requiring clinically closer follow‐up.

## 1 INTRODUCTION

Interatrial block (IAB) is an electrical conduction delay or block between the right and left atrium. IAB is characterized by the presence of a prolonged P‐wave duration that exceeds 120 ms on the 12‐lead surface electrocardiogram (ECG) (Bayés de Luna et al., 2012). The fibrosis of the Bachmann region, the largest interatrial conduction pathway, is thought to be the underlying pathophysiological mechanism of IAB (Alexander et al., 2017). IAB is shown to be associated with atrial fibrillation (AF), ischemic stroke, and increased cardiovascular and all‐cause mortality (Agarwal, Aronow, Levy, & Spodick, 2003; Conde & Baranchuk, 2014; Conde et al., 2015; Tekkesin, Cinier, Cakilli, Hayıroglu, & Alper, 2017; Wu et al., 2016). Therefore, it is important to identify patients who have an increased risk for developing IAB.

Arterial stiffness is a useful prognostic marker for cardiovascular events. It is influenced by age, gender, hypertension (HT), diabetes mellitus (DM), and smoking (Boutouyrie et al., 2002; Mitchell et al., 2010; Willum‐Hansen et al., 2006). It has been shown that increased arterial stiffness may predict cardiovascular events in asymptomatic individuals without overt cardiovascular disease (Maloberti et al., 2018). Pulse wave velocity (PWV) is considered as the main and practical parameter to assess the arterial stiffness.

Obesity represents a global health problem and is associated with cardiovascular risk factors. It is a major predictor of cardiovascular disease (CVD) and mortality (Kannel & Benjamin, 2008; Lavie, Milani, & Ventura, 2009). In addition, obesity is independently related to the development of AF (Wang et al., 2004). It is known that obese patients have an increased arterial stiffness and high risk for developing IAB (Lurbe et al., 2012; Zebekakis et al., 2005).

Obesity is closely linked to cardiac remodeling accompanied by structural and functional abnormalities. Increased left ventricular (LV) mass is seen in obese individuals displaying a positive correlation with BMI. Currently, the mechanisms contributing to these culprit remodeling alterations remain somewhat elusive for obesity although a complex interplay of hemodynamic, neurohormonal, and metabolic factors seems to contribute to oxidative stress, inflammation, apoptosis, hypertrophy, interstitial fibrosis, lipotoxicity, adrenergic, and renin‐angiotensin‐aldosterone overflow. With the effect of these factors, fibrosis increases in cardiac tissue and "obesity cardiomyopathy" develops. The term “obesity cardiomyopathy” was derived mainly based on cardiac functional, morphological, and metabolic abnormalities due to obesity alone, with specific attention to the underlying signaling pathways in increased adiposity. We can observe the finding of increased fibrosis in cardiac tissue as “IAB” and in arterial system as “increased arterial stiffness.”

Overweight is a considerable step in the process leading to obesity. There are no sufficient studies on the effect of cardiomyopathy defined in obese patients about overweight subjects. We thought that it may be useful to examine the cardiovascular involvement in overweight individuals before the development of "obesity cardiomyopathy." In the present study, we aimed to investigate the coexistence of increased arterial stiffness and IAB in overweight subjects.

## 2 MATERIALS AND METHODS

### 2.1 Study population

A total of 110 consecutive overweight patients who were presented for routine checkup and examined at outpatient clinics between April and October 2016 were enrolled. All patients underwent noninvasive ischemia assessment test (treadmill stress test) to exclude obstructive coronary artery disease (CAD). Exclusion criteria were history of atherosclerotic heart diseases (MI, PCI, and CABG), valvular diseases, prior atrial arrhythmias, heart failure, cardiac pacemaker, left ventricular ejection fraction (LVEF) < 55%, bundle branch block, chronic renal and hepatic failure, thyroid and parathyroid dysfunction, peripheral artery disease (ankle‐brachial index < 0.9), uncontrolled hypertension, use of cardiotoxic agent, cardiac depressant drug (B‐blocker, calcium channel blocker, etc.), congenital heart disease, and poor ECG quality. The study was approved by the local ethics committee. All patients signed an informed consent form.

### 2.2 Demographic and echocardiographic evaluation of patients

Body mass index (BMI) was calculated as body weight (kg) divided by height squared (m^2^). Overweight was defined as BMI: 25–29.9 kg/m^2^. Overweight individuals were included in the study. Cigarette smoking was defined as smoking ≥1 packet of cigarettes a day. Blood samples were taken from all participants after 12–14 hr fasting.

All patients underwent two dimensional transthoracic echocardiographic (HD11 XE Ultrasound system, Philips, Canada) evaluation equipped with a 1.5–4.0 MHz transducer. LVEF was obtained by using modified Simpson's method as specified by current guideline of chamber quantification by American Society of Echocardiography (Lang et al., 2015).

### 2.3 ECG analysis

**Figure 1:**
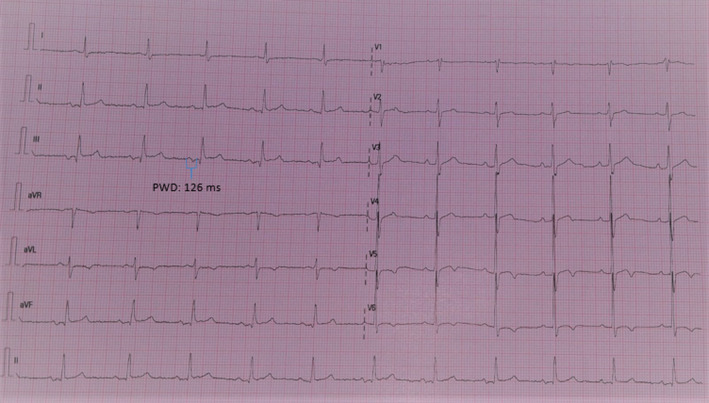
Advanced interatrial block

Surface 12‐lead standard ECGs were recorded from each patient with a 25 mm/s paper speed at 10 mm/mV amplitude (Nihon Kohden Cardiofax M ECG‐1350). ECG images were amplified 8 times, and P‐wave duration was measured blindly by using semiautomatic digital calipers in all 12 leads to acquire the longest duration. All of the measurements were repeated three times, and average values were obtained. The onset of the P‐wave was the point of initial upward or downward deflection from ECG baseline, and the P‐wave endpoint was determined as the point where the waveform returned to baseline (Figure 1). IAB was defined as a P‐wave duration of >120 ms on the 12‐lead ECG (Bayés de Luna et al., 2012). IAB was classified according to the latest consensus article: partial IAB (P‐IAB) as P‐wave duration longer than 120 ms without biphasic morphology in the inferior leads and advanced IAB (A‐IAB) as P‐wave duration longer than 120 ms with biphasic morphology in the inferior leads (Bayés de Luna et al., 2012). Patients with only A‐IAB were included in the present study. All ECG measurements were analyzed by two cardiologists who were blinded to all other data. The intraobserver and interobserver variations for all measurements were <5% and therefore nonsignificant.

### 2.4 Measurement of arterial stiffness

I.E.M. Mobil‐O‐Graph ambulatory blood pressure monitor device and HMS CS analysis software (IEM GmbH) were used to assess the arterial stiffness. This arteriograph system measured pulse wave velocity (PWV), central blood pressure (cBP), augmentation index (Aix), and central pulse pressure (cPP) automatically. Simultaneous brachial measurements provided by the oscillometric Mobil‐O‐Graph arteriograph were taken at 30‐min intervals during the day (9 a.m.‐11 p.m.) and 60‐min intervals at night (11 p.m.‐9 a.m.).

### 2.5 Statistical analysis

SPSS 25.0 (IBM Corp.) program was used for variable analysis. Normally distributed continuous data were expressed as mean ± standard deviation (minimum–maximum). Continuous variables that are not normally distributed were expressed as median (minimum–maximum), and categorical variables were expressed as n and percentages. The normal distribution of the data was evaluated by Lilliefors‐corrected Kolmogorov–Smirnov test and Shapiro–Wilk test, and the variance homogeneity was evaluated by the Levene test. The Independent‐Samples *T* test was used with the Bootstrap results when comparing two independent groups with one according to the quantitative data, and the Mann‐Whitney *U* test was used together with the Monte Carlo results. To compare categorical variables, Pearson chi‐square and Fisher Exact tests were tested using exact results. Correlation analyses were performed to identify the linear correlation among arterial stiffness parameters and P‐wave duration. Variables were examined at 95% confidence level. A *p*‐value < .05 was considered as statistically significant.

## 3 RESULTS

The demographic, echocardiographic, and laboratory characteristics of the study population are presented in Table 1. The present study consisted of 56 patients with IAB [IAB (+) group] and 54 patients without IAB [IAB (−) group]. The mean age of the patients was 54.1 ± 11.5 years, and 53.6% were male. The mean BMI of the patients was 27.8 ± 1.6 kg/m^2^. Of all patients, 66.3% had HT, 37.2% had HLP, 41.8% had DM, and 37.2% were current smokers (Table 1). There were not any significant differences between groups for age, gender, BMI, LVEF, left atrium diameter, smoking, and history of HT, DM, HLP (Table 1).

**Table 1:** Demographic, echocardiographic, and laboratory characteristics

	Total (*n* = 110)	IAB (−) group (*n* = 54)	IAB (+) group (*n* = 56)	*p* Value
Age	54.1 ± 11.5	52.4 ± 10.8	55.2 ± 9.4	.115
Male gender *n *(%)	59 (53.6)	28 (51.8)	31 (55.3)	.205
BMI (kg/m^2^)	27.8 ± 1.6	27.6 ± 2.1	28.1 ± 1.3	.492
P‐wave duration (ms)	109.3 ± 22.4	92.4 ± 6.7	126.8 ± 5.1	<.001
LVEF (%)	65.9 ± 3.6	66.1 ± 3.7	65.8 ± 3.5	.846
Left atrium diameter (mm)	36.7 ± 4	36.1 ± 3.9	37.2 ± 4.1	.062
LVSWT (mm)	9.8 ± 1.5	9.5 ± 1.4	10.2 ± 1.1	.577
Hypertension *n *(%)	73 (66.3)	35 (64.8)	38 (67.8)	.731
Diabetes mellitus *n* (%)	46 (41.8)	22 (40.7)	24 (42.8)	.562
Hyperlipidemia *n *(%)	41 (37.2)	20 (37)	21 (37.5)	.835
Smoking *n *(%)	41 (37.2)	19 (35.1)	22 (39.2)	.094
SBP (mm Hg)	121.5 ± 10.4	120.6 ± 8.7	122.4 ± 9.2	.756
DBP (mm Hg)	77.4 ± 10.2	76.8 ± 9.3	78 ± 9.4	.452
Fasting glucose (mg/dl)	93.2 ± 9.3	91.35 ± 7.5	94.1 ± 8.4	.412
Creatinine (mg/dl)	0.85 ± 0.3	0.86 ± 0.3	0.84 ± 0.4	.818
AST (U/L)	25 (14/ 45)	22 (14/ 42)	26 (16/ 45)	.449
ALT (U/L)	20 (6/ 47)	18 (9/ 47)	23 (6/ 44)	.727
TC (mg/dl)	176.8 ± 34.6	173.2 ± 28.2	179.5 ± 31.3	.396
HDL‐C (mg/dl)	41.6 ± 10.4	42.7 ± 9.8	40.4 ± 10.6	.424
LDL‐C (mg/dl)	119.8 ± 37.1	118.6 ± 35.3	120.9 ± 36.2	.866
TG (mg/dl)	157.2 ± 60.8	145.4 ± 44.9	167.5 ± 49.7	.098
Hemoglobin (g/dl)	15 ± 1.3	15.2 ± 1.1	14.8 ± 1.6	.434
Platelet (×1000) (K/µl)	305 (108/ 435)	285 (108/ 410)	324 (110/ 435)	.562

Abbreviations: BMI, body mass index; DBP, diastolic blood pressure; HDL‐C, high‐density lipoprotein cholesterol; IAB, interatrial block; LDL‐C, low‐density lipoprotein cholesterol; LVEF, left ventricular ejection fraction; LVSWT, left ventricular septal wall thickness; SBP, systolic blood pressure; TC, total cholesterol; TG, triglyceride.

PWV and Aix were significantly higher in IAB (+) group than IAB (−) group (9.34 ± 1.5 vs. 7.86 ± 1.3, *p* < .001; 29.18 ± 11.2 vs. 22.75 ± 10.4, *p* < .001, respectively) (Table 2), and also, positive linear correlation was observed between arterial stiffness parameters and P‐wave duration (*r* = .758 for PWV and P‐wave duration; *r* = .682 for Aix and P‐wave duration, respectively) (Table 3).

**Table 2:** Comparison of arterial stiffness parameters between the groups

	Total (*n* = 110)	IAB (−) group (*n* = 54)	IAB (+) group (*n* = 56)	*p* value
PWV (m/sn)	8.6 ± 2.1	7.86 ± 1.3	9.34 ± 1.5	<.001
Aix (%)	25.96 ± 13.6	22.75 ± 10.4	29.18 ± 11.2	<.001
Central SBP (mm Hg)	116.4 ± 10.2	115.3 ± 8.7	118.9 ± 9.3	.102
Central DBP (mm Hg)	82.4 ± 10.5	80.8 ± 9.5	84.7 ± 8.6	.088
Central pulse pressure (mm Hg)	34 ± 10	34.5 ± 9	34.2 ± 9	.874

Abbreviations: Aix, augmentation index; DBP, diastolic blood pressure; IAB, interatrial block; PWV, pulse wave velocity; SBP, systolic blood pressure.

**Table 3:** Correlation between arterial stiffness parameters and P‐wave duration

	P‐wave duration	
	*r*	*p*
PWV	.758	<.001
Aix	.682	<.001

Abbreviations: Aix, augmentation index; PWV, pulse wave velocity.

There were no significant differences between the groups in terms of central SBP, central DBP, and central pulse pressure values (*p* = .102, *p* = .088, and *p* = .874, respectively) (Table 2).

## 4 DISCUSSION

In the present study, we aimed to determine whether or not there is cardiovascular (CV) involvement in overweight individuals with “arterial stiffness” and “IAB” that are two independent predictors of CV events, before the development of "obesity cardiomyopathy."

In the current study, we demonstrated that increased arterial stiffness was associated with the prolongation of P‐wave duration. There was a positive linear correlation between arterial stiffness parameters (PWV and Aix) and P‐wave duration. To the best of our knowledge, the present study was the first to focus on evaluating the relationship between the presence of IAB and arterial stiffness in overweight people.

"Obesity cardiomyopathy" is a complex interplay of direct and indirect pathophysiologic factors related to obesity. Obesity is an independent risk factor for CAD, and strongly associated with HT, and DM, which indirectly leads to the development of ischemic, hypertensive, and diabetic cardiomyopathy, respectively. Since obesity cardiomyopathy has multiple and interacting pathways, it is not possible to mention a direct mechanism.

Obesity has different effects on cardiovascular structures and functions. Obesity increases cardiac preload, resulting in compensatory remodeling (Abel, Litwin, & Sweeney, 2008), and induces the expression of paracrine hormone with endovascular effects that may also alter atrial pressures and preload conditions (Martin, Qasim, & Reilly, 2008). Also, obesity has been reported to directly drive electrophysiologic remodeling by altering the myocardial matrix secondary to adipose‐derived hormones (Schram & Sweeney, 2008). This remodeling induces interatrial dyssynchrony, and electromechanical dysfunction of the left atrium with increased pressure, atrial dilatation, endothelial dysfunction, and finally atrial fibrosis. Atrial fibrosis is considered to be the main pathophysiological mechanism leading to IAB.

Arterial stiffness is a strong predictor of all‐cause mortality and is often associated with other cardiovascular risk factors, such as smoking, obesity, HT, HLP, and DM. Several prior studies showed that oxidative stress, production of free radicals, and neuroendocrine changes contributed to cardiovascular damage and early arterial stiffening (Cecelja & Chowienczyk, 2009; Naka et al., 2012). Obesity is an important risk factor for functional and structural damage to the arterial wall, resulting in early arterial stiffness. Stiffness of vascular smooth muscle cells, extracellular matrix remodeling, adipose tissue inflammation, and immune cell dysfunction contribute to the development of arterial stiffness in obesity (Aroor, Jia, & Sowers, 2018).

Although electromechanical atrial dysfunction was known in obese subjects, there was not enough information about overweight people. Sun, Zhou, Ye, Wu, and Sun (2019) aimed to assess the independent associations of obesity and hypertension with IAB. Their study evidenced a relatively higher prevalence of IAB in subjects with hypertension and elevated BMI. Liu et al. (2010) investigated the effect of obesity on P‐wave parameters in a Chinese population. They measured maximum P‐wave duration (Pmax), minimum P‐wave duration (Pmin), and P‐wave dispersion (Pd) using a 12‐lead electrocardiogram. Pd was defined as the difference between Pmax and Pmin (Pd = Pmax −Pmin). They showed that obesity was associated with increased Pmax and Pd, and increased prevalence of IAB, parameters that have been associated with atrial fibrillation. However, they reported no satisfactory information about the presence and effects of IAB in overweight subjects. Also, they found that compared with controls, left atrial diameter increased in the obese group. In the present study, we did not find any difference between groups in terms of left atrium diameter in overweight subjects. This finding is important in terms of showing that there may be atrial involvement without increased left atrial diameter.

Increased arterial stiffness may predict cardiovascular events in asymptomatic individuals without overt cardiovascular disease. In several studies, increased PWV values in healthy normotensive individuals indicated subclinical cardiovascular damage. Li, Wang, and Liu (2017) evaluated the relationship between overweightness, obesity, and arterial stiffness. They demonstrated that arterial stiffness, a recognized marker of cardiovascular risk, is increased in obese/overweight subjects without overt cardiovascular diseases. Therefore, assessment of arterial stiffness may identify patients at risk at an early stage.

It is well known that the presence of cardiovascular risk factors (older age, HT, DM, etc.) increases both the likelihood of IAB and arterial stiffness. However, in the present study, there were not any significant differences between IAB (+) and IAB (−) groups for age, gender, HT, and DM. As mentioned earlier, there are many direct or indirect mechanisms in the pathophysiology of obesity cardiomyopathy, and it is not correct to hold only known factors (age, HT, DM, etc.) in the formation and outcome of this cardiomyopathy.

The main strength of our study was the coevaluation of arterial stiffness and IAB in overweight individuals, which are strong predictors and prognostic factors for CV events. Considering that IAB indicates “cardiac atrial involvement” and increased arterial stiffness indicates “vascular involvement”; according to our results, it should be examined especially for CV events in overweight individuals with IAB and increased arterial stiffness.

### 4.1 Limitations

This study had several limitations. First, the cross‐sectional design of our study was unable to evaluate the longitudinal associations and distinguish causality between arterial stiffness and IAB. Since these patients do not have any follow‐up, we do not have the information regarding the new onset AF among IAB (+) group. Second, the present study had the limited number of patients (110). Finally, further studies are needed to determine the real diagnostic and prognostic role of these atrial and arterial abnormalities in the clinical management of overweight subjects.

## 5 CONCLUSION

In conclusion, the current study was the first to focus on evaluating the relationship between the presence of IAB and arterial stiffness in overweight subjects. PWV and Aix were significantly higher in IAB (+) group than IAB (−) group, and also, positive linear correlation was observed between arterial stiffness parameters and P‐wave duration.

## CONFLICT OF INTEREST

The authors declare that there is no conflict of interest.
